# Photoactive metabolite mediated photodynamic therapy of Rhabdomyosarcoma cell lines using medicinal plants and Doxorubicin co-treatments

**DOI:** 10.1186/s12906-024-04575-2

**Published:** 2024-07-15

**Authors:** Sumbal Javaid, Irfan Zia Qureshi, Ahmat Khurshid, Tayyaba Afsar, Fohad Mabood Husain, Muhammad Khurshid, Janeen H. Trembley, Suhail Razak

**Affiliations:** 1 Animal Physiology Laboratory, Department of Animal Sciences, Quaid-i-Azam University, Islamabad, Pakistan; 2 Biophotonics and Photonanomedicine Research laboratory (BPRL), Department of Physics and Applied Mathematics, Pakistan Institute of Engineering and Applied Sciences (PIEAS), Islamabad, Pakistan; 3 Department of Community Health Sciences, College of Applied Medical Sciences, King Saud University, Riyadh, KSA 11451 Saudi Arabia; 4 Department of Food Science and Nutrition, College of Food and Agriculture Sciences, King Saud University, Riyadh, Saudi Arabia; 5 School of Biochemistry and Biotechnology, University of the Punjab, Lahore, Pakistan; 6 Minneapolis VA Health Care System Research Service, Minneapolis, MN USA; 7 Department of Laboratory Medicine and Pathology, University of Minnesota, Minneapolis, MN USA; 8 Masonic Cancer Center, University of Minnesota, Minneapolis, MN USA

**Keywords:** Photosensitizer, Rhabdomyosarcoma cancer, Photodynamic therapy, Absorption spectroscopy, Combination index, Medicinal plants

## Abstract

**Background:**

Medicinal plant-mediated combinational therapies have gained importance globally due to minimal side effects and enhanced treatment outcomes compared to single-drug modalities. We aimed to analyze the cytotoxic potential of each conventional treatment i.e., photodynamic therapy (PDT), chemotherapy (doxorubicin hydrochloride; Dox-HCl) with or without various concentrations of medicinal plant extracts (PE) on soft tissue cancer Rhabdomyosarcoma (RD) cell line.

**Methods:**

The Rhabdomyosarcoma (RD) cell line was cultured and treated with Photosensitizer (*Photosense* (AlPc4)), Chemo (Dox-HCl), and their combinations with different concentrations of each plant extract i.e., *Thuja occidentalis*, *Moringa oleifera*, *Solanum surattense*. For the source of illumination, a Diode laser (λ = 630 nm ± 1 nm, P_max_ = 1.5 mW) was used. Photosensitizer uptake time (∼ 45 min) was optimized through spectrophotometric measurements (absorption spectroscopy). Drug response of each treatment arm was assessed post 24 h of administration using 3-(4, 5-dimethyl-2-thiazolyl)-2, 5- 5-diphenyl-2 H- tetrazolium bromide (MTT) assay.

**Results:**

PE-mediated Chemo-Photodynamic therapy (PDT) exhibited synergistic effects (CI < 1). Moreover, Rhabdomyosarcoma culture pretreated with various plant extracts for 24 h exhibited significant inhibition of cell viability however most effective outcomes were shown by low and high doses of *Moringa oleifera* compared to other plant extracts. Post low doses treated culture with all plant extracts followed by PDT came up with more effectiveness when compared to all di-therapy treatments.

**Conclusion:**

The general outcome of this work shows that the ethanolic plant extracts (higher doses) promote the death of cancerous cells in a dose-dependent way and combining Dox-HCl and photo-mediated photodynamic therapy can yield better therapeutic outcomes.

## Background

Cancer is the leading cause of death among children and adolescents worldwide with approximately 300,000 reported cases annually [1]. Rhabdomyosarcoma (RMS) is a soft tissue sarcoma, commonly found in children and adolescents, and children with metastatic Rhabdomyosarcoma show poor prognosis [2]. Distant metastasis is the major cause of death in this disease. Rhabdomyosarcoma cell lines exhibit significant cellular heterogeneity, which is important for understanding the complexities of cancer biology and the development of effective treatments. This heterogeneity allows researchers to study various aspects of cancer, such as the role of specific proteins, the effects of different treatments, and the interactions between different cell types [3]. Other modalities for RMS treatment include chemotherapy, radiotherapy, and surgery. Despite all these established treatments, the metastatic RMS disease prognosis has not improved in decades, so there is a need to improve the treatment strategies for the disease [4].

Photodynamic therapy (PDT) is a non-invasive light-based treatment [5]. PDT can be used to treat cancer as a single treatment or in combination with conventional treatment modalities. In PDT, cell damage occurs due to the production of reactive oxygen species (ROS) through various mechanisms (apoptosis, necroptosis, autophagy, and necrosis) and can also occur by induction of immunological responses [6]. In the recent era instead of the use of synthetic chemotherapeutic drugs, the use of natural substances and herbal drugs is gaining popularity for cancer treatment as they are environmentally sustainable and lack major side effects [7]. The success of plants for cancer treatment is due to the presence of bioactive compounds such as flavonoids, phenolic compounds, vitamin C (ascorbic acid), a-tocopherol, b-carotenoids, etc.). Botanical extracts show inhibitory properties on cell survival and proliferation by enhancing apoptosis and targeting key signaling pathways.

Nowadays combinational drug therapies (administration of more than one anticancer drug) are widely used as a cancer treatment [8, 9]. Therapeutic regimens employing combinations of drugs are increasingly favored for treating diverse ailments, notably cancer. These combinations leverage the synergistic or additive effects of distinct pharmaceutical agents to eliminate cancer cells while minimizing additional adverse effects [10]. Conventional chemotherapeutic drugs such as Doxorubicin (DOX), Cisplatin, and methotrexate (MTX) are clinically proven to combat different cancer types [11]. DOX acts by halting DNA replication leading to inhibition of the proliferation and generation of reactive oxygen (ROS) species which leads to tumor destruction mechanism. Combinatorial approaches such as chemo-immunotherapy, chemo-photodynamic therapy (PDT), chemo-radiotherapy, and chemo-phytotherapy are garnering attention due to their enhanced therapeutic outcomes, including synergistic or additive effects, mitigation of multidrug resistance, and attenuation of drug-induced toxicity via diverse cellular mechanisms [10].

*Moringa oleifera* (Mo) has been used as a traditional medicinal source for centuries. All parts of the *Moringa oleifera* tree (e.g., pods, seeds, and leaves) have been used to cure many diseases and are known as ‘‘miracle vegetables’’ [12, 13]. The *Moringa oleifera* tree is a high beta-carotene source of fats, proteins, iron, potassium, vitamin C, and other nutrients which make it highly nutritious. *Moringa oleifera* contains a variety of active phytoconstituents, including alkaloids, protein, quinine, saponins, flavonoids, tannins, steroids, and glycosides. Its leaves are also known to be a rich source of polyunsaturated fatty acids, specifically omega-3 and omega-6 (ω-6) in the form of linoleic and α-linolenic acid. Additionally, leaves are a good source of polyphenols and polyfavonoids, which are antioxidants and perhaps anticancer substances [14, 15]. *Moringa oleifera* has been examined for its different biological activities including anti-atherosclerotic, immune-boosting, antioxidant, anti-cardiovascular diseases, antiviral antimicrobial, anti-inflammatory properties, and tumor-suppressive effects [16]. However, fewer studies have described the anti-cancerous activity of *Moringa oleifera* leaves [17].

*Thuja occidentalis* is generally known as white cedar or Arborvitae. It is traditionally used to cure various diseases such as bronchial distress urinary infections, psoriasis, uterine carcinoma, skin diseases, spongy tumour blood, and rheumatism. It has been reported as an anti-metastatic agent [18]. *Thuja occidentalis* is rich in terpenoids, flavonoids, and tannin-like components. Leaf extract of *Thuja occidentalis* phytochemical compounds present are tannic acid, as well as polysaccharides and proteins glycoproteins or polysaccharides [19]. *Thuja occidentalis* leaf extract contains polyphenols alkaloids along with thujone which is considered an important phytochemical for medicinal uses. The ethanolic leaf extract of *Thuja* is reported to cure different diseases including cancer [20].

*Solanum surattense* belongs to the family Solanaceae and is commonly known as nightshade or yellow berried nightshade. Since ancient times, it has been an important medicinal herb in the sub-continent. It is found as a weed in the wasteland [21]. *Solanum surattense* has significant therapeutic applications. Anti-cancerous effects of *Solanum surattense* extracts are attributed to the presence of flavonoids such as apiginene, quercitin, fisatin, luteolin, terpenoids, alkaloids, steroids, glycosides etc. [21] which make it a potent inhibitor of cancer cell proliferation [22]. The anti-cancerous efficiency of fruit extract of *Solanum surattense* is reported for human lung cancer cell lines (HOP-62) and leukemic (THP-1) cell lines [23].

Current work was aimed to provide an ample study on the measure of cytotoxic effects of different concentrations of three natural plant extracts (*Moringa oleifera*,* Thuja occidentalis*,* and Solanum surattense*), chemo drug (Dox-HCl) and their effect on the efficacy of phthalocyanine (*Photosense*^®^) mediated Photodynamic therapy.

## Methods

### Photosensitizer

In the current study second-generation PS, phthalocyanine (*Photosense*^®^) is used as a photosensitizer. Working dilution of the photosensitizer from the stock solutions was freshly made on the day of experiment.

### Cellular uptake time of photosensitizer (PS)

To measure the time of cellular uptake of the phthalocyanine freshly prepared working solutions for different *Photosense* concentrations were made by diluting the stock solution with media. Briefly, RD cells were cultured into flat bottom 96 well plates and were treated with different concentrations of *Photosense* (25 µM, 50 µM, 100 µM, 150 µM). To assess the cellular uptake of *Photosense*^®^, we utilized a microplate reader, specifically the BioTek ELX 800 model, to measure the absorption of the compound. This involved quantifying the optical density (OD) of light at 630 nm. By monitoring absorption at various time intervals, we pinpointed the peak absorption of *Photosense*^®^, indicating the ideal duration for cellular uptake. This analysis establishes the optimal time frame at which *Photosense*^®^ is most effectively taken up by the cells, offering crucial insights for future experiments and enhancing the compound’s utilization.

### Subculturing of cell line

The Rhabdomyosarcoma (RD) cell line (received from the National Institute of Health, Islamabad) was cultured in a 25cm^2^ tissue culture flask (Nunc Wiesbaden, Germany) in Hank’s minimum essential medium (HMEM) supplemented with 5% Fetal Bovine Serum (FBS). To achieve adherent monolayer cells were grown in a favorable environment at 37 °C in a laminar flow hood. Microscopic images showed clear branch-like morphology of culture cells, which showed that cells are in monolayer and fully stretched and can occupy more space if allowed to split further.

### Plant collection and preparation of extracts

*Moringa oliefera* and *Solanum surattense* were harvested at an age of about 6 months and *Thuja occidentalis* was harvested at an age of about 2 years. Fresh leaves of all three plants (*Moringa oleifera*,* Thuja occidentalis*,* and Solanum surattense*) were collected from district Poonch of Azad Kashmir and identified by Professor Dr Mir Ajab from the Department of Plant Sciences, Quaid-e-Azam University, Islamabad. The collected leaves were then washed thoroughly with distilled water and then shade dried (7 days), ground into a fine powder then stored in an airtight jar separately till further use. For the preparation of plant extract (P.E), the powdered sample (30 gm.) of every plant was soaked in 300 ml (1; 10) of 70% ethanol (ETOH) for 72 h. Using a glass rod mixtures were [24] stirred every 24 h and then filtered using a Whatman filter paper. A rotary evaporator (*SENCO*^®^) at a temperature of 40–45ºC for 6 h at 200 rpm was used to concentrate the filtrates and then the filtrate was dried in an oven at 50ºC. The plant extracts were weighed and stored in glass storage vials at 4 °C For plants the stock solutions (5 mg/ml) were prepared by solubilizing extract (25 mg) with DMSO (5 ml).

### Plant extract and chemotherapy treatment

The study protocol was approved by research board committee of Pakistan Institute of Engineering and Applied Sciences (PIEAS, Ref.no. DMS/INVR/PRL/DPAM/2012/03–22). RD cells were seeded for 24 h to achieve 70–80% confluency and then treated with two different concentrations of *Moringa oliefera*,* Solaum surrettense*, and *Thuja occidentalis* (50,150 µg/ml) [25], Dox-HCl and *Photosense* (50,150µM). All dilutions were made fresh, we used 10 µl from each stock solution (50 µM, 150µM) and added it in the culture well with 200 µl culture media. Initially, RD cell culture was exposed/ treated with low and high doses of all plant extracts, Dox-HCl, and *Photosense* (PS) individually without light exposure. Secondly, confluent RD culture was then treated in di-combination with high doses of plant extracts (150 µg/ml) and chemo (150 µM). In another di combination high dose of plant extracts (150 µg/ml) and a low dose of *Photosense* (50 µM) were administered the combinational treatment was carried out for all three plants separately. The PS was added in each experiment group 45 min before carrying out the MTT assay.

To evaluate the tri combination the cell culture was pre-treated with plant extracts (50 µg/ml) in low dose and a high dose of Dox-HCl (150 µM) followed by a low dose of PS (50 µM) PDT.

In another treatment group culture was pre-treated with a high dose of plant extracts (150 µg/ml) a low dose of Dox-HCl (50 µM) and a low dose of PS (50 µM) was added 45 min before PDT was carried out.

In the last group of the treatment arm high dose of plant extracts (150 µg/ml) and a low dose of Dox-HCl (50 µM) were added to the culture and PS (150 µM) mediated PDT was carried out.

### Photodynamic treatment

**Fig. 1:**
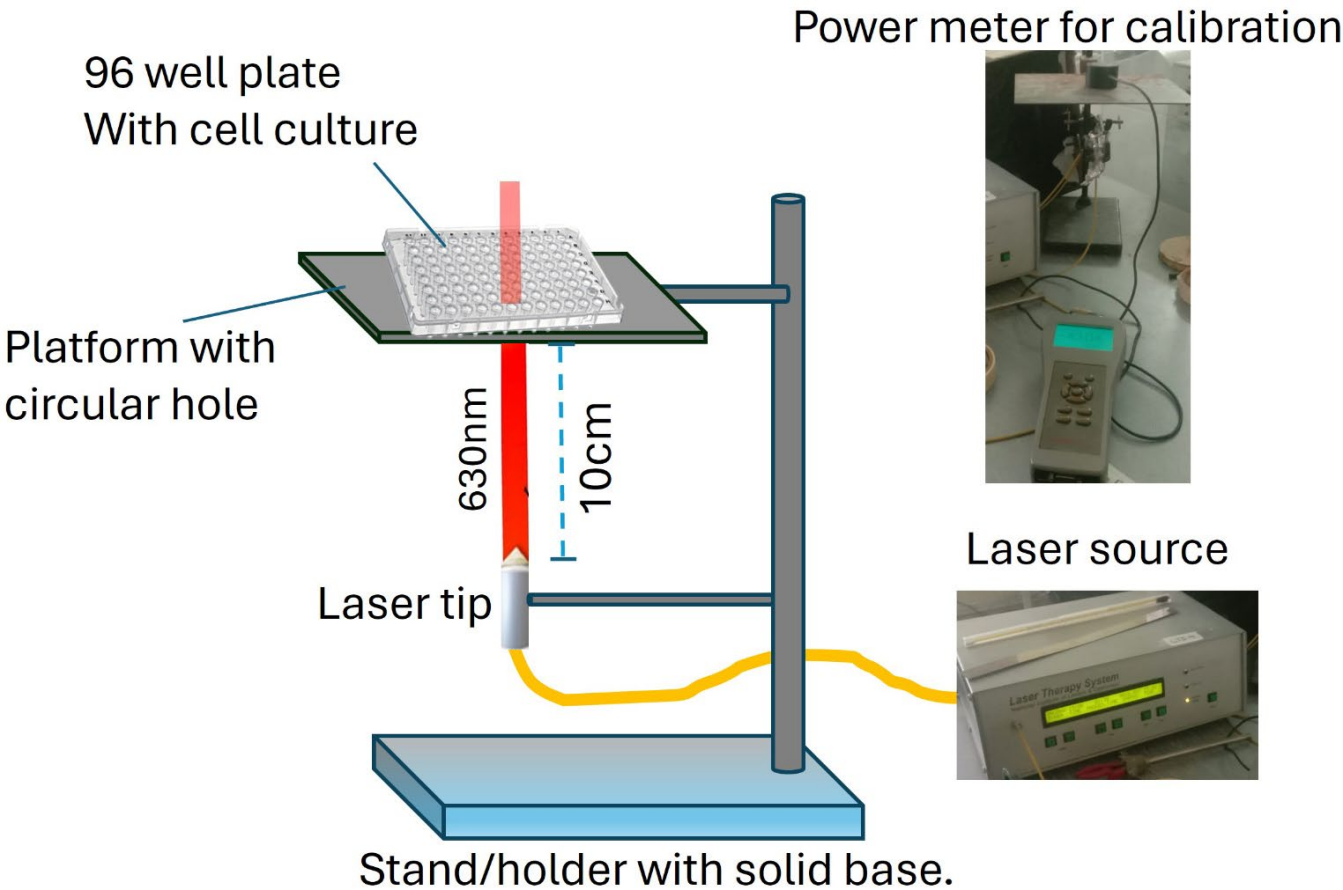
Photodynamic therapy setup

For plant extract mediated chemo photodynamic (Tri therapy) therapy RD cells were grown in a 96-well plate with a cell density of 1 × 10^5^ cells per well and then incubated with the treatment. Then the culture was exposed to both high of PS according to the treatment groups explained above. After this, the cells were irradiated with light of 630 nm wavelength using a clinical semiconductor laser diode the system used is LPhT-630/675-01-BIOSPEC, Russia. Before irradiation, the laser source was calibrated by using a power meter (Thor Labs, Germany). The laser diode fiber tip was positioned 10 cm below the 96-well plate with round bottom wells (Fig. 1). The plate rested on a horizontal platform featuring a circular aperture matching the area of the wells. Using a power meter, the intensity was measured at 10 mW. This value was used to calculate the exposure time required for the desired light energy dose 2J/cm^2^ dose which was 66 s for 2J after irradiance, cell culture media was then replaced with a fresh 2% minimum essential medium and then the culture was incubated for 24 h. Energy density was measured using the equation.

$$\text{Energy} \,\text{density} \,\left(\text{J}/\text{cm}^{2}\right)\hspace{0.17em}=\hspace{0.17em}\text{irradiance} \,\left(\text{mW}/\text{cm}^{2}\right) \times \text{time} \left(\text{s}\right)$$

Throughout the experiment, the untreated cells (cells + culture growth media) were used as a control group to exclude the possible natural cell death of RD. HMEM Media was used as vehicle control. Any possible cell death may be excluded through the normalization of data. The toxicity of all the selected therapeutic modalities individual or in combination (di and Tri) was evaluated through colorimetric assay (MTT) [26].

### Cell viability

To study the in vitro cytotoxicity of plant extracts and drugs, the effect of various treatments on the cell viability of RD cells was estimated by MTT assay. The MTT reagent, sourced commonly from Sigma Aldrich, is utilized as it is reduced by mitochondrial dehydrogenase enzymes to form purple formazan [26, 27]. RD cells were cultured and 96 well plates were.

prepared with a cell density of 1 × 10^5^ cells per well and incubated for 24 h at culturing conditions. Cells were treated with plant extracts (50 µM, 150 µM), PS (50 µM, 150 µM) and Dox-HCl (50 µM, 150 µM) according to the experimental treatment groups. This was done by taking 10 µl from each stock dilution (P.E, DoxHCl and PS) and adding it to each well already containing 200 µl culture media. Cells were further incubated at culturing conditions depending on the optimized uptake time of the drugs and were then washed thrice with PBS, followed by adding media. After that MTT working solution was added to each well and the plate was incubated for 4 h. The Media was then aspirated and a solubilizing agent 100 µl was added to each well. Plates were shaken for 5 minutes and then optical density was recorded for each well at 630 nm by using a microplate reader (Biotec ELX). Quantitative assessment of cell viability entails measuring the absorbance attributed to formazan production by viable cells. The percentage of viable cells in the cell population at each concentration was calculated using the following formula:

$$\% {\text{ }}Viability{\text{ }}=\frac{{{A_{treated}} - {A_{empty}}}}{{{A_{control}} - {A_{empty}}}} \times 100$$

where ${A}_{treated }$ is the mean absorbance of the treated cell, ${A}_{empty}$ is the mean absorbance of empty wells, and ${A}_{control}$ is the absorbance of control cells that are not exposed to any drug or light [27].

### Cellular morphological study

Following post-incubation treatments, culture responses, including morphological alterations (such as roundness, elongation, swelling, and density variations) and intercellular physical interactions, were documented. Utilizing a microscope (XDS-2, Optika Microscope Italy) equipped with an integrated camera, visual data were captured and transferred to a computer for documentation and analysis.

### Drug interaction modeling

The obtained cytotoxicity data were analyzed using the CI model. This analysis was performed using the CompuSyn software program, which is available online. The CI model is a widely used method for evaluating the combination effects of multiple drugs or treatments. The CompuSyn software program is a computational tool designed to assess the synergistic, additive, or antagonistic effects of different combinations of drugs or treatments. It provides a.

quantitative measure of the combined effect based on dose-response data. The CI value may be < 1,> 1 or can be = 1 to explain data as synergistic, antagonistic, or additive [28].

### Statistical analysis

Data are expressed by mean ± SEM (*n* = 3). One-way analysis of variance (ANOVA) followed by Tukey’s test was applied to calculate the statistical changes among different experimental groups using GraphPad Prism 9 software. The level of significance was set at *p* < 0.05.

## Results

### Cellular uptake time of photosensitizer (PS)

**Fig. 2:**
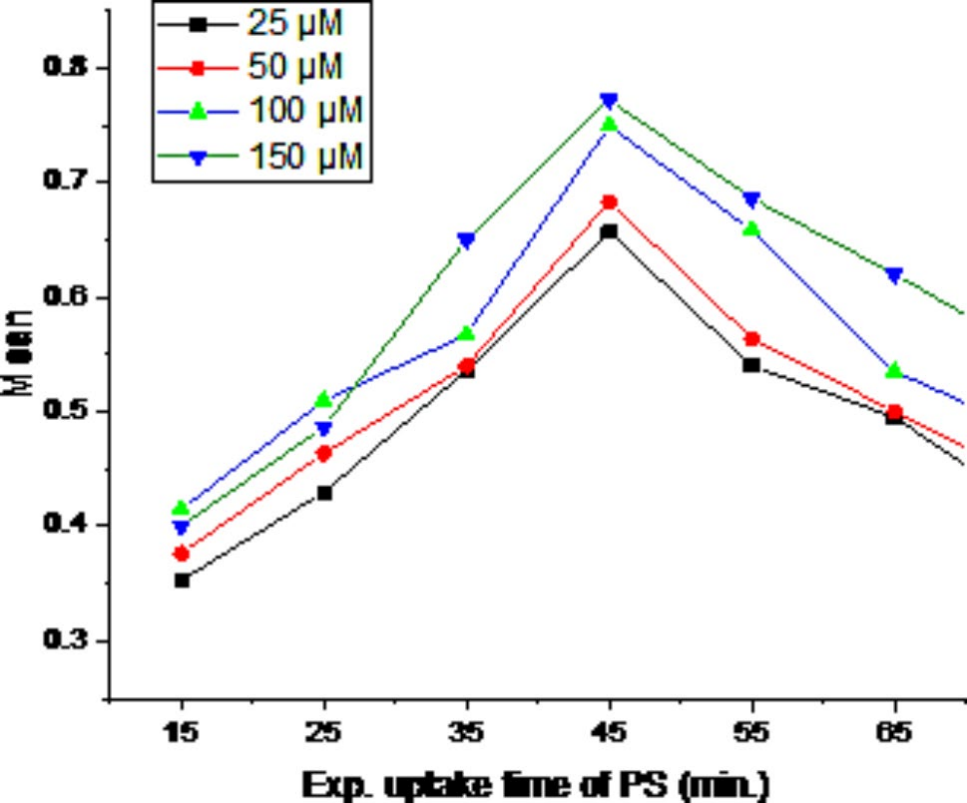
Optimal time for maximum uptake of *Photosense* in RD cell culture

To determine the optimal duration of the uptake of PS by the RD cell lines, cells were treated with various doses (25 µM, 50 µM, 100 µM, 150 µM) of photosensitizer. Afterwards, the optical density of the cells was determined using a BioTek plate reader at various time intervals. The maximum absorption corresponding to the maximum uptake time for the photosensitizer was 45 min as shown in Fig. 2. This result is indicative of the preferential intake of PS by the RD cells with varying time intervals.

### Different treatment arms dose-response cytotoxicity

**Fig. 3:**
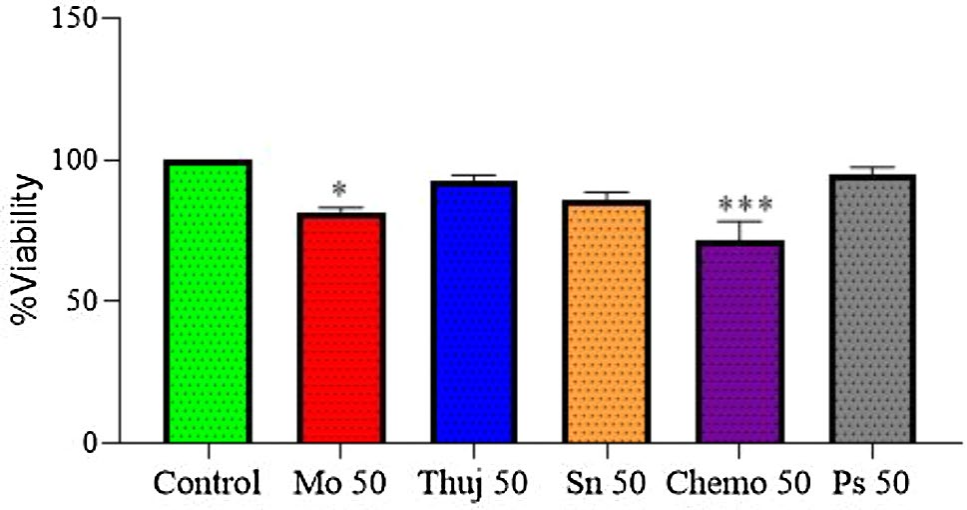
Individual cell viability of lower doses of different treatment arms (*Moringa oleifera; MO*,* Thuja occidentalis; Thuja*,* and Solanum surattense; Sn at 50* µg/ml); chemo drug (Dox-HCl) and photosensitizer phthalocyanine (µM)) on Rhbodmayosarcoma cell culture. Data indicated the results of three independent experiments (*n* = 3). Asterisks * and *** show significant difference at *p* < 0.05 and *p* < 0.001 *versus the* control group. Data was analyzed by one-way ANOVA followed by Tukey’s multiple comparison test

It was observed that administration of a lower dose of *Moringa oleifera*, *Thuja occidentalis*, and *Solanum surattense* (50 µg/ml) to the cell culture showed less cell inhibitory potential when compared with the control group. Photosense at a concentration of 50 µM did not exhibit induced cytotoxic potential at a lower tested dose. However, Chemo (Dox-HCl) at a concentration of 50 µM treatment showed inhibition of cell viability (30%). The experiment was run with no light exposure (@ 0 J /cm^2^) (Fig. 3).

**Fig. 4:**
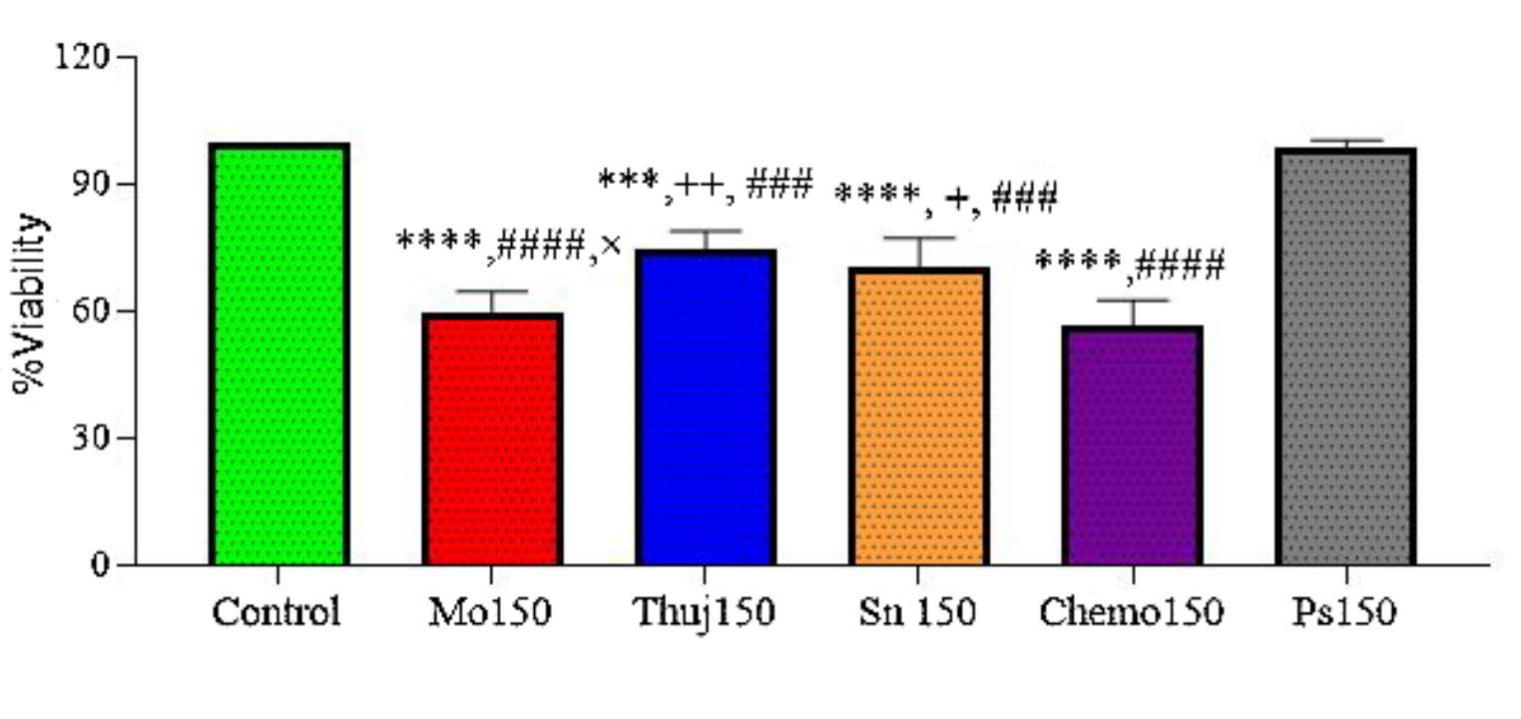
Cytotoxicity of *Moringa oleifera*, *Thuja occidentalis*, and *Solanum surattense* at 150 µg/ml concentration. Chemo drug and phthalocyanine (*Photosense*) were administered at 150 µM doses. Data indicated the results of three independent experiments (*n*=3). Asterisks *** and **** show significant difference at *p*<0.001 and *p*<0.0001 *versus the* control group, whereas + and ++ showed significant difference at *p*<0.05 and *p*<0.01 versus the chemo 150 group. ### and #### indicated significant difference at *p*<0.001 and *p*<0.0001 *versus* PS 150 treatment group. × showed a comparison at *p*<0.05 between different plant extracts at high doses. Data was analyzed by one-way ANOVA followed by Tukey’s multiple comparison test. Mo: *Moringa oleifera*, Thuj: *Thuja occidentalis*, Sn: *Solanum surattense*, Ps: Photosense, Chemo: Doxorubicin chemotherapy

**Fig. 5:**
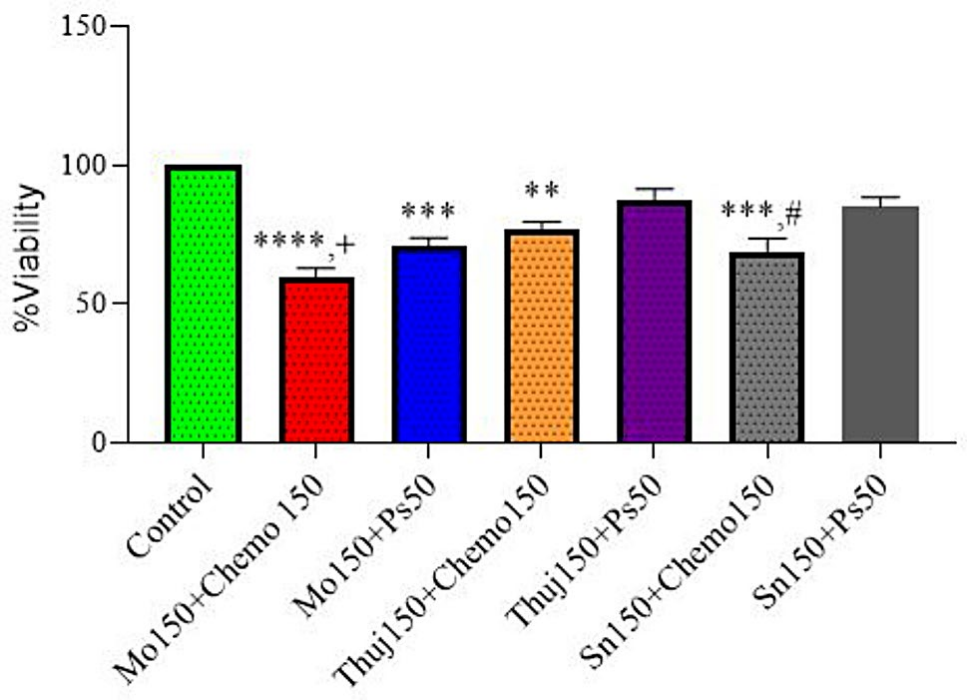
Effect of Di-combination of higher concentration (150 µg/ml) of Plant extracts, chemo (Dox-HCl), and photosensitizer on cell proliferation of Rhabdomyosarcoma cells. PS incubated for 45 min post 24 h of Plant & chemo drug. Data indicated the results of three independent experiments (*n* = 3). Asterisks **, ***, and **** shows significant differences at *p* < 0.01, *p* < 0.001, and *p* < 0.0001 *versus the* control group, whereas + shows a significant difference of Mo 150 + Chemo150 group at *p* < 0.05 *versus* Thuj150 + Chemo 150 group. # indicated significant difference of Sn 150 + Chemo 150 group at *p* < 0.05 versus Sn 150 + PS 50 group. Non-significant difference was noticed between Mo150andChemo150 *versus* Mo150 + Ps50 and Thuj150andChemo150 *versus* Thuj150 + Ps50 groups. Data was analyzed by one-way ANOVA followed by Tukey’s multiple comparison test. Mo: *Moringa oleifera*, Thuj: *Thuja occidentalis*, Sn: *Solanum surattense*, Ps: *Photosense.* Chemo: Doxorubicin chemotherapy

**Fig. 6:**
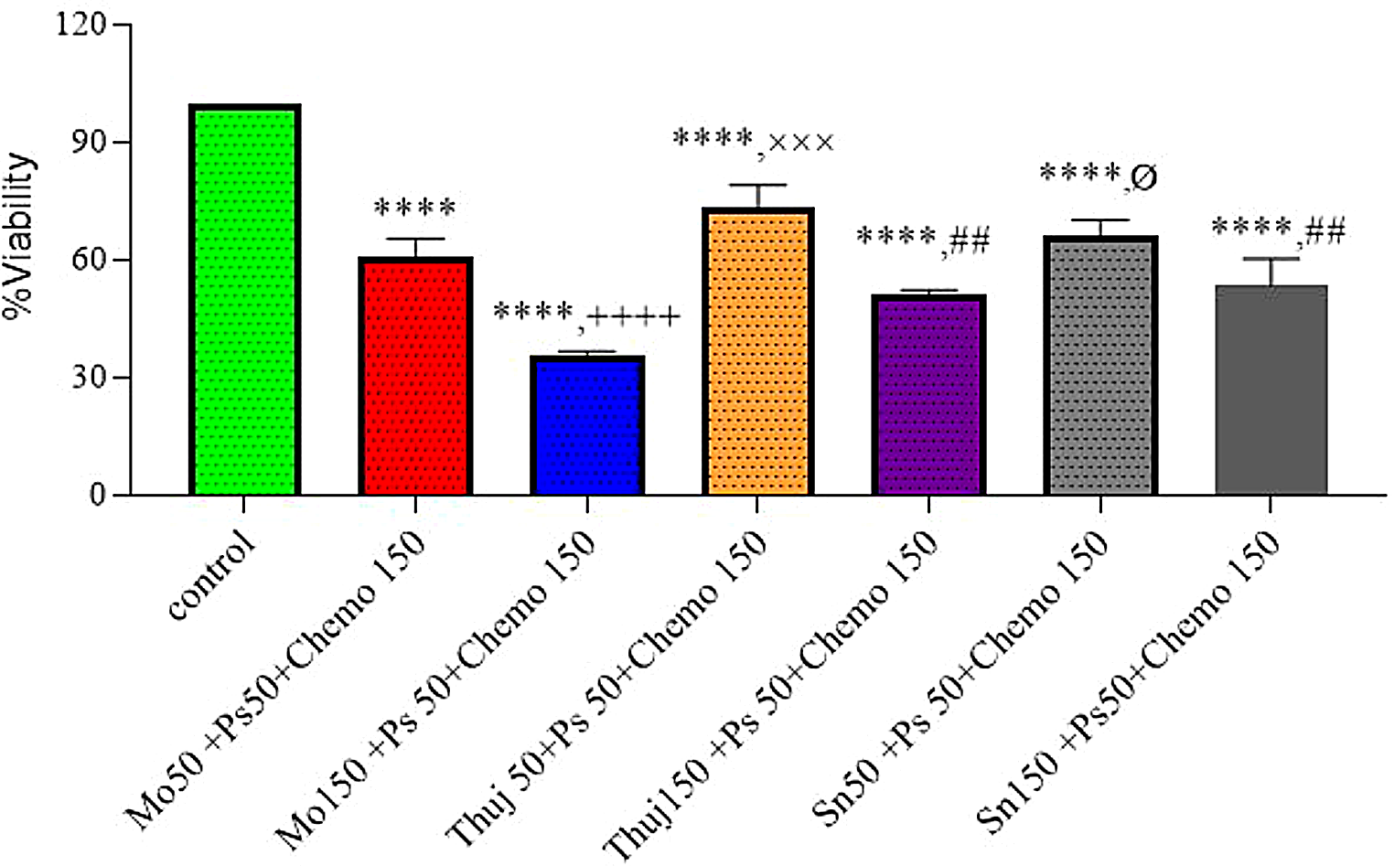
Tri-combination with a low dose of PS (50 µM), a higher dose of Chemo (150µM), and different doses of plants (50,150 µg/ml) followed by *Photosense*^®^-mediated PDT. Data indicated the results of three independent experiments (*n* = 3). Asterisks **** show a significant difference at *p* < 0.0001 *versus the* control group. ++++ shows significant difference at *p* < 0.0001 of Mo 50 + Ps50 + Chemo150 treatment group *versus* Mo150 + PS 50 + Chemo150 treatment groups. ××× indicated significant difference at *p* < 0.001 of Thuj50 + Ps50 + Chemo150 versus Thuj 150 + PS 50 + Chemo150 treatment groups. ## indicated significant difference at *p* < 0.01 of Mo150 + Ps 50 + Chemo 150 treatment group *versus* Thuj150 + PS 50 + Chemo150 and Sn150 + Ps50 + Chemo150 treatment groups. Ø indicated a significant difference at *p* < 0.05 of Sn50 + Ps50 + Chemo150 *versus* Sn150 + PS 50 + Chemo150 treatment groups. Data was analyzed by one-way ANOVA followed by Tukey’s multiple comparison test. Mo: *Moringa oleifera*, Thuj: *Thuja occidentalis*, Sn: *Solanum surattense*, Ps: Photosense, Chemo: Doxorubicin

In another treatment arm of mono therapy, the percent cell viability of RD cell culture, when exposed to the higher doses of plant extracts (150 µg/ml) *(Moringa oleifera*,* Thuja occidentalis*,* Solanum surattense*), photosense and Dox-HCl (150 µM), were recorded. For the case of PS, a mild-cell inhibitory response was expected which is a basic criterion of good and ideal photoactive drugs in the absence of light. Cellular viability in response to PS remains independent of doses (50 µM,150 µM). *Moringa oleifera showed 60% cell viability*,* Thuja occidentalis showed 75% viability* and Solanum *surattens* showed 70% as Dox-treated cells exhibited 56% cell viability (Fig. 4). A comparison of three plant extracts indicated more potent (*p* < 0.05) cell inhibition induced by *Moringa oleifera* at 150 µg/ml dose compared to *Thuja occidentalis* and Solanum *surattense* treatment group *Moringa oleifera* (59 ± 2.7), and higher concentration of Dox-HCl (56 ± 2.9) showed significant cell inhibition compared to the control, furthermore, the cell inhibitory efficacy of *Moringa oleifera (*150 µg/ml) was equivalent to the Dox-HCl group as indicated by a non-significant difference among both groups. In di-combination treatment higher doses of all three plants (150 µg/ml) with Dox-HCl (150 µM) exhibited less percentage of cell viability. In combination with a higher dose of Dox-HCl *Moringa oleifera* showed 59% cell viability, *Thuja occidentalis* showed 75% and *Solanum surattense showed 68% cell viability* The treatment arms with a higher dose of plant extracts but a low dose of Ps showed more cell viability close to the values of mono treatment of high doses that attributes to the reason that PS without light is non-cytotoxic (Fig. 5). Higher doses of all the combinations came out to be more synergistic. Compared to di-combination when tri-combination was assessed it was observed that all the treatment arms (PE, Chemo, and PS-PDT, diode laser, 630 nm, 2 J/cm^2^) showed significant cell inhibition in RD cell culture (Fig. 6). In the tri-drug combination, *Moringa oleifera* and *Solanum surattense* with Dox-HCl showed more synergistic effects as compared to *Thuja occidentalis* extract (Table 1). It may be concluded that both the selected doses (higher and lower) of *Moringa oleifera* in the presence of Dox-HCl (150 µM) and PS (50 µM) showed around 40% and 75% reduction in cell viability respectively. In this treatment the combination of *Moringa oleifera* 150 µg/ml, Dox. HCl 150µM and PS 50µM showed more significant results compared to *Thuja occidentalis (tri combination)* and *Solanum surattense* (tri combination).

These results showed that higher doses of plant extracts are more significant in tri-combination therapy compared to lower doses of plant extract. Moreover, higher doses of plant extracts alone are less effective as compared to when administered in combination with Dox-HCl. and PDT. The di- and tri-combinations response to the in-vitro cell culture in the presence of a higher dose of Dox-HCl (150 µg/ml) is presented in tabulated form (Table 1).

**Table 1:** RD cell culture response to Di- and tri-combinations for lower and higher doses of selected treatment arms

Dox-HCl@150 µM		Photosense @ 50 µM	
Plant extracts (PE)150 µg/ml	Cell viability	Plant extracts (PE) 150 µg/ml	Cell viability
**Di-Combinations**			
*Moringa oleifera*	∼ 59%	*Moringa oleifera*	∼ 70%
*Thuja occidentalis*	∼ 76%	*Thuja occidentalis*	∼ 87%
*Solanum surattense*	∼ 68%	*Solanum surattense*	∼ 85%
**Tri-Combinations**			
*Moringa oleifera*	∼ 60%	*Moringa oleifera*	∼ 35%
*Thuja occidentalis*	∼ 73%	*Thuja occidentalis*	∼ 50%
*Solanum surattense*	∼ 65%	*Solanum surattense*	∼ 53%

**Fig. 7:**
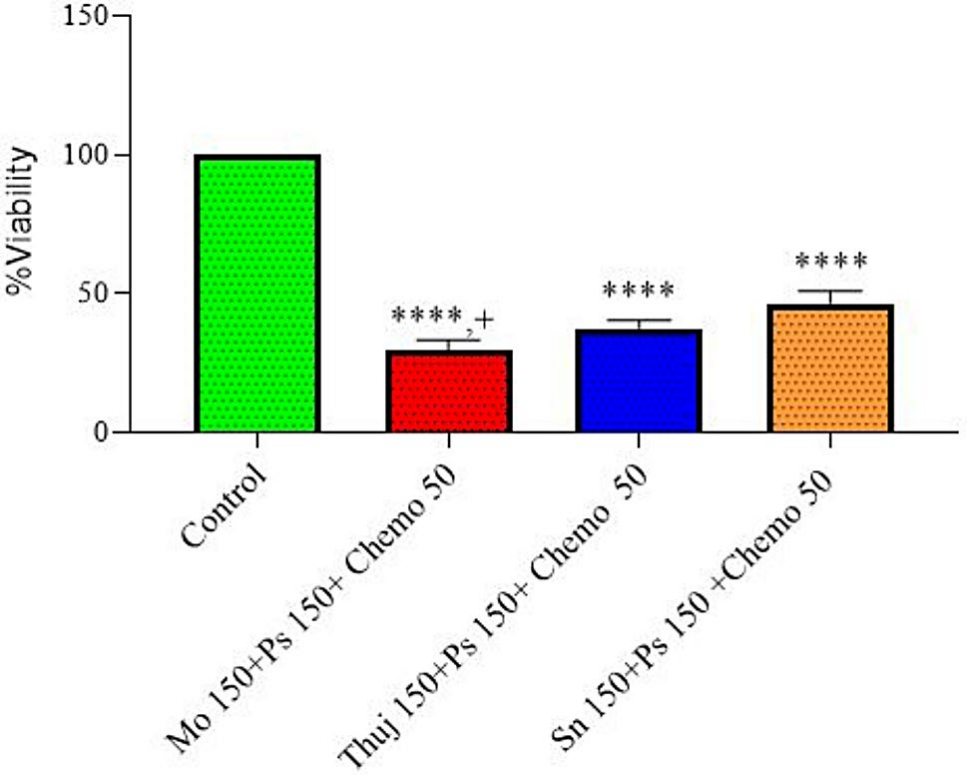
Plant extracts (*Moringa oleifera*,* Thuja occidentalis*,* and Solanum surattense*) 150 µg/ml chemo 50 µM, (Dox-HCl) and photosensitizer150 µM, individual cytotoxicity on rhabdomyosarcoma cell culture. Data indicated the results of three independent experiments (*n* = 3). Asterisks **** showed a significant difference at *p* < 0.0001 *versus the* control group. + showed significant difference at *p* < 0.05 of Mo150 + PS150 + Chemo50 group *versus* Sn150 + PS150 + Chemo50 groups

In the last treatment group deduced from the above results higher concentration of all plants that are 150 µg/ml with a low dose of 50 **µM** of chemo, to minimize chemo adverse effects were administered in combination with PS-PDT @150**µM**, 2J/cm^2^ evaluated as shown in Fig. 7 all the treatment arms showed a significant reduction of cell viability. Among all three plant extracts *Moringa oleifera* exhibited the most significant results (28 ± 3.1) It was observed that PE showed a synergistic response which may be attributed to chemo cum PDT-mediated production of reactive oxygen species (ROS). The overall MTT effect of photosense-mediated plant-chemo-PDT for RD culture was dose-dependent.

### A cellular morphological study using inverted visible light microscopy

**Fig. 8:**
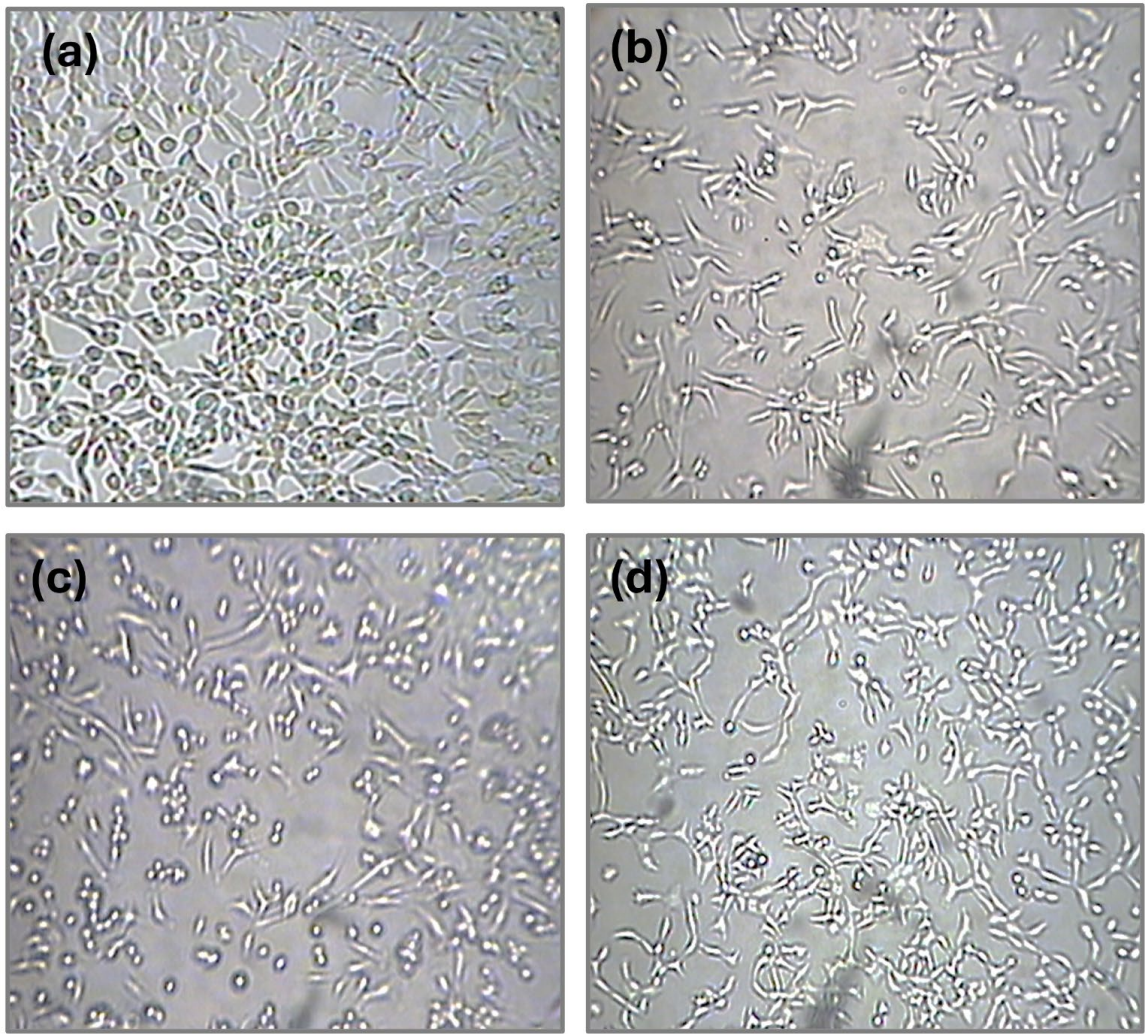
Morphological changes (% cell survival) of various treatment groups in the presence of light. **(a)** Control (100%), **(b)***Moringa oleifera* (MO, 28%), **(c)***Solanum surattense* (Sn, 37%) **(d)***Thuja occidentalis* (Thuj, 46%). PS: photosense, Chemo: Doxorubicin chemotherapeutic

Images captured on a CCD-coupled inverted microscope also showed that the combinational treatment reduces the density of cells that are viable (Fig. 8). It was observed that when the Plant extracts were administered in the presence of photosensitizer and chemo drug morphology of cell culture changes i.e., round and swelled cells, which also suggests the apoptotic mode of cell Death [29]. Post Tri-combination images of plant extracts (150 µg/ml) with low dose Dox-HCl (50**µM**) with PDT@ Ps (150**µM**) showed considerable morphological changes, indicating cell death. The treatment groups exhibited apoptotic features such as detached and rounded cells, while no morphological changes were observed in the control group.

### Combination index (Ci) by the chau-talalay method

Combinational index (CI) values were calculated by using Compusyn software [30]. The results supported the hypothesis of P.E-assisted chemo-PDT. It was observed that strong, moderate, and marginal synergistic effects may result from the presented combinations. From the calculated CI values (Table 2) for di-combinations, it may be concluded that synergistic effects are observed with combination of Dox-HCl (150 µM) and *Moringa oleifera* (150 µg/ml) as compared to *Solanum surattense* and *Thuja occidentalis*. From the CI values it is evident that the plant extract (150 µg/ml) in combination with chemo drug low dose (50 µM) in the presence of photodynamic therapy@ 2 J showed more synergism exhibiting decreased CI values for *Moringa oleifera*, *Solanum surattense*, and *Thuja occidentalis* in sequential order.

**Table 2:** Combinational index values for different combinations of PE, Chemo, and PS

Samples	Combination index (CI)		
	Di therapy Dox-HCl (150 µM)	Tri therapy	
		**Dox-HCl** (150 µM) **photosence** (50 µM) (2J/cm^2^)	**Dox-HCl** (50 µM) **Photosence** (150 µM) (2J/cm^2^)
***Moringa oleifera*** **(**150 µg/ml)	0.57	0.41	0.23
***Thuja occidentalis*** **(**150 µg/ml)	0.77	0.56	0.55
**Solanum** **(**150 µg/ml)	0.69	0.49	0.34
**Dox-HCl** (50 µM) + **AlPc**_**4**_ (150 µM)		0.81	0.63

Based on the values of CI presented in the above table may conclude that the di- & tri- combination in the presence of plant extracts are synergistic (< 1) in nature.

## Discussion

Nature, as a reservoir of herbal resources, harbours unexplored pharmaceutical compounds that offer significant benefits to human health. These substances have been employed since ancient times to address both cancerous and noncancerous ailments. Studies focusing on plant extracts reveal that these natural products induce cancer cell death through alterations in the cell cycle, apoptosis, and modulation of various signaling pathways [31]. Chemotherapeutic drugs such as Dox-HCl are prospective anti-cancer drugs with therapeutic benefits but that exhibit side effects. Drug combinations have become a common treatment option for a variety of diseases, including cancer [32]. The two most important qualities of any successful treatment are low toxicity to normal cells and great efficiency. The advantage of therapeutic combinations is that the various medications can kill cancer cells in an additive or synergistic way, without causing any additional side effects, for a range of cancer types [33]. Recently, synergistic interactions have been discovered. Plant-based compounds, such as flavonoids and polyphenols, are effective against a variety of cancers and can be used with chemotherapy drugs [34, 35]. It is pertinent to use natural products in conjunction with chemo agents to reduce toxicity, delay or lessen drug resistance, and potentiate synergistic therapeutic effects [36].

In our research, we examined the combined effects of three different plants on Dox-HCl and PS-PDT to determine if using a combination of plant extracts with low doses of DOX and PS-PDT on RD cells could be an effective cancer treatment.

In our study, *Moringa oleifera* over the administered range of concentrations (lower doses) seems to be non-cytotoxic [37]. Higher doses of *Moringa oliefera* alone and in combination with Dox-HCl and with PDT showed significant cell death is in accordance with work done earlier on other cancer cell lines and also in combination with the chemo drug and PDT [38]. It has shown significant cytotoxicity due to the occurrence of bioactive compounds and their metabolites [24]. Bioactive compounds such as isopropyl, isothiocyanate, D-allose, cetene octadecene, tetradecane, octadecanoic acid, and palmitic acid are present exclusively in the leaves extract of *Moringa* [39]. Due to the immense ability of cancer cells to proliferate, anticancer drugs are needed to target these cells and prevent them from growing. It’s interesting to note that *Moringa oliefera* carries out a variety of biological functions and even targets many proteins and macromolecules to slow the proliferation of cancer cells [40]. It has glycosides, which are highly effective against cancer and are capable of inducing apoptosis these results are relevant to published data as shown by Berkovich et al. according to this publication extracts from *Moringa oliefera* leaves have been reported to suppress the growth of pancreatic cancer cells [38].

*Solanum surattense* also has anticancer activity attributing to the presence of phytochemicals it [41]. In our study RD cell culture showed mild cytotoxicity over the lower dose of *Solanum surattense* where as high doses alone showed moderate cytotoxicity and combinational treatment with Dox and PDT showed more significant cytotoxicity. It has been reported that cytotoxicity associated with *Solanum surattense* may be attributed to the presence of a second metabolite commonly known as solamargine, a steroidal alkaloid [21, 42]. This explains a dose-dependent cytotoxicity of *Solanum surattense* leave extracts and its cytotoxicity increase in combination with Dox-HCl which has been reported earlier for *Solanum nigrum* ethanolic extract in MCF-7 cell line [43].

*Thuja occidentalis* showed notably more viable cells in combinational treatment with Dox and PDT as compared to the other two plant extracts. Studies have indicated that *Thuja occidentalis* acts as an anticancer agent in a number of ways according to S.Saha et al. *Thuja occidentalis* induce apoptosis of cells of mammary epithelial carcinoma [44]. It also lowers the level of cytokines (IL-1β, TNF-α) [45]. It also down-regulates IL-6 which is an indicator of tumor reduction factor. Our results support the cytotoxicity initiated by the plants over the selected doses and in a dose-dependent manner on RD cells and it is evident that a combinational approach is critical and beneficial for the treatment of cancer. Studies have shown that plant extracts exhibit the properties of imbibition on cell survival and proliferation by increasing apoptotic activity [46] by targeting various signaling pathways like PI3K/AKT, ERK/MAPK, and NF-κB. They also exhibit anticancer activity by reducing levels of anti-apoptotic factors such as BCL-2 and BCL-XL, while activating the pro-apoptotic elements like BAX BAK, BID, and caspases. The plant extracts raise reactive oxygen species (ROS), which triggers caspases, cleaves PARP-1, and causes P53 to trigger cancer cell apoptosis [47].

All the treatment groups in our study which have been exposed to chemo drugs showed less viability of cells. Cytotoxicity associated with Dox-HCl is reported in Chemo treated arms, which may be due to the reason that Doxorubicin on cancer cells works by two different mechanisms firstly by intercellular intercalation which disrupts topoisomerase-II-mediated DNA repair; and secondly due to free, radical’s generation, which damages proteins. DNA, and cellular membranes [48]. Doxorubicin can also generate cytotoxic reactive oxygen species to exert cellular damage selection of the range of concentration and it may not upregulate or downregulate cellular death mediating proteomic pathways [49].

All the treatment arms that are exposed to photodynamic therapy showed significant results this can be attributed When there is molecular oxygen present, the activated photosensitizer sets off a series of photochemical and photobiological reactions that cause highly reactive oxygen species (ROS), mostly singlet oxygen, to act on intracellular organelles resulting in cancer cell death [50, 51]. This has been observed that PS only (without light) has shown higher viability which means more cell replication and higher viabilities contributed to the preferential accumulation of intracellular localization of PS in mitochondria and lysosomes. It has been reported previously that higher viability in the presence of PS contributed to higher metabolic activities in the absence of suitable light. [52]. It was expected that in the absence of light, there would be no photochemical reactions that may lead to the oxidation of cellular organelles. The intracellular localization of PS is a vital parameter in defining the cell death mechanism and efficacy of PDT. Phthalocyanine is a stabilized PS localized in lysosomes, mitochondria & cytoplasm (intracellular organelles). On light activation of PS, these organelles destroy the membrane through ROS production, which causes cell death [53, 54]. PS mitochondrial localization induces apoptosis and either apoptosis or necrotic response is induced due to the lysosomal localization of PS [55]. PDT-produced intracellular ROS can directly kill tumour cells by inducing necrosis and/or apoptosis through a range of signaling pathways in combination with caspases, apoptosis-inducing factors, and Bcl-2 family members [56]. Also, phthalocyanine-mediated PDT causes cell cycle arrest at G_0_/G_1_ followed by inhibition of cell growth and cell death of human cervical carcinoma cells [57]. Dox-HCl strived cell death via induction of senescence-like phenotype (SLP) and down-regulation of different proteins when lower doses were administered [58]. Dox-HCl in the presence of PS-PDT encourages singlet oxygen-dependent cytotoxic effects [59]. Outcomes of phase II clinical studies have shown considerable potential for the doxorubicin combination with additional chemo medicines for treating metastatic Rhabdomyosarcoma [60, 61]. Photodynamic therapy has also been shown to have synergistic effects in the treatment of advanced pancreatic cancer [62]. This study hypothesizes that combining Dox-Photosense-PDT and natural active chemicals may improve the treatment results by minimizing toxicity associated with a high dosage of chemo drugs [10]. Apoptosis, inhibition of Topoisomerase I, ROS production in cancer cells, and other biological pathways leading to cell death are triggered when cancer cells are treated with a combination of agents, such as PDT, chemotherapy drugs, and plant extracts (a mixture of many chemicals) [63] ROS production in cancer cells is one of the mechanisms underlying the synergistic cytotoxicity of combination therapy involving plant, chemo drug, and photodynamic therapy, thereby enhancing the cytotoxic effect of the treatment [64]. because many plants have protective effects thereby reducing side effects of chemo drugs.

Various studies have shown that the most successful cancer treatments are combinational therapies that attempt to target multiple non-overlapping cellular pathways and also minimize the high-dose chemotherapy adverse effects [65]. Researchers observed that a dual extended chemo-PDT combinational therapy method minimized high-dosage chemotherapy-related adverse effects while maintaining performance status The efficacy of the individual modalities, plant extract, chemo drug, and photodynamic therapy was enhanced when combined in the same treatment regime which is also supported by other researchers [32, 66].

It is well documented in several published research articles that PDT alone is not sufficient to produce effective therapeutic outcomes due to several reasons such as cancer cells adopt alternate molecular pathways to respond to the damaged mitochondria, and its efficacy increases with combination therapy, secondly, not all the mitochondria get shut down in response to the intracellular uptake of drugs, etc. Therefore, it is normally a need for an alternate approach to bring in a second or third treatment modality to combat several up-regulated pathways that cancer cells activate to survive and metastasize to distant parts [67]. Our results support the positive collaborative effects of the combination of plant and synthetic drug-mediated PDT thereby reducing the possible side effects of the drug. In comparison to mono treatment, a combination of PDT and chemo intensifies the synergistic effects to incite increased cytotoxicity of cancer cells thereby reducing the possible side effects of the drug [64, 68].

### Limitations of the study

The in-vivo cancer model to study the response of these combinational therapeutic modalities has not been studied in the current investigation. The expected challenge is the bio-distribution of these drugs intracellular, which may be addressed with the encapsulation in FDA-approved PLGA nanocarriers. The second challenge may be the drug release profile keeping up-regulation of molecular pathways. These limitations may lead to the synthesis of multilayered controlled drug-released nanocarriers. Extensive in-vivo study (Toxicity, bioavailability, biocompatibility, yield of therapeutic outcome) may decide the translation into the clinic, for which the author will be much more excited.

## Conclusion

This work shows that chemo-PDT therapeutic outcomes may be enhanced in the presence of plant extract (tri-combination). It has been concluded that chemo-PDT therapeutic outcome may be enhanced in the presence of plant extract, the tri-combination, which is favoured as compared to di-combination. This modality may be a potential candidate for the best therapeutic outcome when low doses are desired to be administered in the cancer cell culture. Alone Low-dose treatment is not potent against cancer cells, while higher doses cause cytotoxicity, but combining different therapies produces a promising result. Findings of the present work showed that the extracts stimulate cancer cell death in a dose-dependent manner, and better therapeutic outcomes can be achieved in combination with the Dox-HCl, and the phthalocyanine-mediated photodynamic therapy. Among all three tested extracts of plants, *Moringa oleifera* turns out to be the most effective in plant-mediated chemo photodynamic therapy.

## Data Availability

All the relevant data has been provided in the manuscript. Datasets used and/or analyzed during the current study are available from the corresponding author upon reasonable request.

## Abbreviations

PDT: Photodynamic therapy
Dox-HCl: Doxorubicin hydrochloride
RMS: Rhabdomyosarcoma
DMSO: Dimethyl sulfoxide
PS: Photosensitizer
PBS: Phosphate buffered saline
ETOH: Ethanol
HMEM: Hank’s Minimum Essential Medium
FBS: Fetal bovine serum
OD: Optical density
CI: Combinational index
Mo: Moringa oleifera
Thuj: Thuja occidentalis
Sn: Solanum surattense

## References

[1] Hawkins DS, Spunt SL, Skapek SX (2013). Children’s Oncology Group’s 2013 blueprint for research: soft tissue sarcomas on behalf of on behalf of the COG Soft Tissue Sarcoma Committee. Pediatr Blood Cancer.

[2] Kim JR, Yoon HM, Koh KN, Jung AY, Cho YA, Lee JS (2017). Rhabdomyosarcoma in children and adolescents: patterns and risk factors of distant metastasis. Am J Roentgenol.

[3] Yamasaki (2014). The Use of Animal models for Cancer Chemoprevention Drug Development. Bone.

[4] Mahmood R, et al. Vitamin D3-assisted chemo-photodynamic therapy of rhabdomyosarcoma cancer cells for effective treatment. Laser Phys Lett. 2018;15(12). 10.1088/1612-202X/aae219.

[5] Zhou Z, Zhang L, Zhang Z, Liu Z. Advances in photosensitizer-related design for photodynamic therapy, *Asian J. Pharm. Sci*, vol. 16, no. 6, pp. 668–686, Nov. 2021, 10.1016/J.AJPS.2020.12.003. 10.1016/j.ajps.2020.12.003 PMC8737425 35027948

[6] Palumbo G (2007). Photodynamic therapy and cancer: a brief sightseeing tour. Expert Opin Drug Deliv.

[7] Shi H, Sadler PJ (2020). How promising is phototherapy for cancer?. Br J Cancer.

[8] Dos Santos AF, De Almeida DRQ, Terra LF, Baptista MS, Labriola L. Photodynamic therapy in cancer treatment - an update review. J Cancer Metastasis Treat. 2019;2019. 10.20517/2394-4722.2018.83.

[9] Ahmed S, et al. Apoptosis induced by luteolin in breast cancer: mechanistic and therapeutic perspectives. Phytomedicine. March, 2019;59. 10.1016/j.phymed.2019.152883. 10.1016/j.phymed.2019.152883 30986716

[10] Mokhtari RB et al. Combination therapy in combating cancer SYSTEMATIC REVIEW: COMBINATION THERAPY IN COMBATING CANCER BACKGROUND, *Oncotarget*, vol. 8, no. 23, pp. 38022–38043, 2017, [Online]. Available: www.impactjournals.com/oncotarget. 10.18632/oncotarget.16723 PMC5514969 28410237

[11] Ahmed F, Ijaz B, Ahmad Z, Farooq N, Sarwar MB, Husnain T. Modification of miRNA Expression through plant extracts and compounds against breast cancer: Mechanism and translational significance, *Phytomedicine*, vol. 68, no. August 2019, p. 153168, 2020, 10.1016/j.phymed.2020.153168. 10.1016/j.phymed.2020.153168 31982837

[12] Khalafalla MM (2010). Active principle from Moringa oleifera Lam leaves effective against two leukemias and a hepatocarcinoma. Afr J Biotechnol.

[13] Abd-Rabou AA, Abdalla AM, Ali NA, Zoheir KMA (2017). Moringa oleifera root induces cancer apoptosis more effectively than leave nanocomposites and its free counterpart. Asian Pac J Cancer Prev.

[14] Paikra BK, Gidwani B. Phytochemistry and Pharmacology of Moringa oleifera Lam, pp. 194–200, 2017. 10.3831/KPI.2017.20.022 PMC5633671 30087795

[15] Saini RK (2016). Phytochemicals of Moringa oleifera: a review of their nutritional, therapeutic and industrial significance. 3 Biotech.

[16] Kou X, Li B, Olayanju JB, Drake JM, Chen N. Nutraceutical or pharmacological potential of Moringa oleifera Lam., *Nutrients*, vol. 10, no. 3, Mar. 2018, 10.3390/NU10030343. 10.3390/nu10030343 PMC5872761 29534518

[17] Balogun TA, Buliaminu KD, Chukwudozie OS, Tiamiyu ZA, Idowu TJ. Anticancer potential of Moringa oleifera on BRCA-1 gene: Systems Biology. Bioinform Biol Insights. 2021;15. 10.1177/11779322211010703. 10.1177/11779322211010703 PMC8842389 35173424

[18] Caruntu S, Ciceu A, Olah NK, Don I, Hermenean A, Cotoraci C, editors. Thuja occidentalis L. (Cupressaceae): Ethnobotany, Phytochemistry and Biological Activity, *Molecules*, vol. 25, no. 22, pp. 1–15, 2020, 10.3390/molecules25225416. 10.3390/molecules25225416 PMC7699608 33228192

[19] Caruntu S, Ciceu A, Olah NK, Don I, Hermenean A, Cotoraci C, editors. molecules Thuja occidentalis L. (Cupressaceae): Ethnobotany, Phytochemistry and Biological Activity, 10.3390/molecules25225416. 10.3390/molecules25225416 PMC7699608 33228192

[20] Khan AS. Medicinally important trees. Med Important Trees. 2017;1–309. 10.1007/978-3-319-56777-8.

[21] Tekuri SK, Pasupuleti SK, Konidala KK, Amuru SR, Bassaiahgari P, Pabbaraju N (2019). Phytochemical and pharmacological activities of Solanum surattense burm. f.-A review. J Appl Pharm Sci.

[22] Mukhtar M (2020). Anticancer agents from Solanum Surattense. Front Chem Sci.

[23] Imran M et al. September., Luteolin, a flavonoid, as an anticancer agent: A review, *Biomed. Pharmacother*, vol. 112, no. 2018, 2019, 10.1016/j.biopha.2019.108612. 10.1016/j.biopha.2019.108612 30798142

[24] Farooq B, Koul B, Mahant D, Yadav D (2021). Recently, synergistic interactions have been discovered. Plant-based compounds, such as flavonoids and polyphenols, have been shown to be effective against a variety of cancers and can be used with chemotherapy drugs. Plants.

[25] Bhadresha K, Thakore V, Brahmbhatt J, Upadhyay V, Jain N, Rawal R (2022). Anticancer effect of Moringa oleifera leaves extract against lung cancer cell line via induction of apoptosis. Adv Cancer Biol - Metastasis.

[26] Bahuguna A, Khan I, Bajpai VK, Kang SC. MTT assay to evaluate the cytotoxic potential of a drug, pp. 115–8, 2017, 10.3329/bjp.v12i2.30892.

[27] Aziz B, et al. Study of synergistic effects of Ficus Carica leaves extract mediated chemo-photodynamic therapy on rhabdomyosarcoma cells. Photodiagnosis Photodyn Ther. Dec. 2021;36:102565. 10.1016/J.PDPDT.2021.102565. 10.1016/j.pdpdt.2021.102565 34614426

[28] Hegazy MEF et al. November., Cytotoxicity of 40 Egyptian plant extracts targeting mechanisms of drug-resistant cancer cells, *Phytomedicine*, vol. 59, no. 2018, p. 152771, 2019, 10.1016/j.phymed.2018.11.031. 10.1016/j.phymed.2018.11.031 31055230

[29] Zanini C et al. Nov., Inhibition of heat shock proteins (HSP) expression by quercetin and differential doxorubicin sensitization in neuroblastoma and Ewing’s sarcoma cell lines., *J. Neurochem*, vol. 103, no. 4, pp. 1344–1354, 2007, 10.1111/j.1471-4159.2007.04835.x. 10.1111/j.1471-4159.2007.04835.x 17680992

[30] www.combosyn.com.

[31] Iqbal J, et al. Plant-derived anticancer agents: a green anticancer approach. Asian Pac J Trop Biomed. Dec. 2017;7(12):1129–50. 10.1016/J.APJTB.2017.10.016.

[32] Fuel M, et al. Antioxidant and antiproliferative potential of ethanolic extracts from Moringa oleifera, Tropaeolum tuberosum and Annona cherimola in colorrectal cancer cells. Biomed Pharmacother. Nov. 2021;143. 10.1016/J.BIOPHA.2021.112248. 10.1016/j.biopha.2021.112248 34649364

[33] Bhatia K, Bhumika, Das A. Combinatorial drug therapy in cancer - new insights. Life Sci. Oct. 2020;258:118134. 10.1016/J.LFS.2020.118134. 10.1016/j.lfs.2020.118134 32717272

[34] Du GJ, Song ZH, Lin HH, feng Han X, Zhang S, ming Yang Y (2008). Luteolin as a glycolysis inhibitor offers superior efficacy and lesser toxicity of doxorubicin in breast cancer cells. Biochem Biophys Res Commun.

[35] Mittal A, Tabasum S, Singh RP (2014). Berberine in combination with doxorubicin suppresses growth of murine melanoma B16F10 cells in culture and xenograft. Phytomedicine.

[36] Kumari P, Luqman S, Meena A (2019). Application of the combinatorial approaches of medicinal and aromatic plants with nanotechnology and its impacts on healthcare. DARU J Pharm Sci.

[37] Barhoi D, Upadhaya P, Barbhuiya SN, Giri A, Giri S. Aqueous Extract of Moringa oleifera Exhibit Potential Anticancer Activity and can be Used as a Possible Cancer Therapeutic Agent: A Study Involving In Vitro and In Vivo Approach, *J. Am. Coll. Nutr*, vol. 40, no. 1, pp. 70–85, 2021, 10.1080/07315724.2020.1735572. 10.1080/07315724.2020.1735572 32191153

[38] Rehman A, Minhas AS. Green route synthesis of copper oxide nanoparticles against MCF-7 cell line (Breast Cancer). NUST J Nat Sci. 2021;30:6(1).

[39] Sci-Hub. | Moringa oleifera leaves crude aqueous extract down-regulates of BRCA1, mta-1 and oncogenes c-myc and p53 in AsPC-1, MCF-7 and HTC-116 cells. Food Bioscience, 43, 101221 | 10.1016/j.fbio.2021.101221.https://sci-hub.mksa.top/10.1016/j.fbio.2021.101221 (accessed Dec. 13, 2021).

[40] Jung IL (2014). Soluble extract from Moringa oleifera leaves with a new anticancer activity. PLoS ONE.

[41] Kumar P (2021). A review on the pharmaceutical activity of Solanum surattense. GSC Adv Res Rev.

[42] Fu R (2019). Solamargine inhibits gastric cancer progression by regulating the expression of lncNEAT1_2 via the MAPK signaling pathway. Int J Oncol.

[43] Dewi D, Putri P, Rivanti E, Prima Istiaji R, Meiyanto E. Solanum nigrum Ethanolic Extract (SNE) Increases Cytotoxic Activity of Doxorubicin on MCF-7 Cell, *Indones. J. Cancer Chemoprevention*, vol. 12, no. 2, pp. 67–73, Oct. 2021, 10.14499/INDONESIANJCANCHEMOPREV12ISS2PP67-73.

[44] Saha S (2014). Contribution of the ROS-p53 feedback loop in thuja-induced apoptosis of mammary epithelial carcinoma cells. Oncol Rep.

[45] Chang LC (2000). Bioactive constituents of Thuja occidentalis. J Nat Prod.

[46] Banerjee S, Nau S, Hochwald SN, Xie H, Zhang J (2023). Anticancer properties and mechanisms of botanical derivatives. Phytomedicine Plus.

[47] Mansoor S (2022). Reactive oxygen species in plants: from source to Sink. Antioxidants.

[48] Kciuk M (2023). Doxorubicin—An Agent with multiple mechanisms of Anticancer Activity. Cells.

[49] Meredith A-M, Dass CR. Increasing role of the cancer chemotherapeutic doxorubicin in cellular metabolism. J Pharm Pharmacol. Jun. 2016;68:729–41. 10.1111/jphp.12539. 10.1111/jphp.12539 26989862

[50] Robertson CA, Evans DH, Abrahamse H. Photodynamic therapy (PDT): a short review on cellular mechanisms and cancer research applications for PDT. J Photochem Photobiol B Biol. Jul. 2009;96(1):1–8. 10.1016/J.JPHOTOBIOL.2009.04.001. 10.1016/j.jphotobiol.2009.04.001 19406659

[51] Mroz P, Yaroslavsky A, Kharkwal GB, Hamblin MR. Cell death pathways in photodynamic therapy of Cancer, pp. 2516–39, 2011, 10.3390/cancers3022516. 10.3390/cancers3022516 PMC3729395 23914299

[52] Crous A, Dhilip Kumar SS, Abrahamse H. Effect of dose responses of hydrophilic aluminium (III) phthalocyanine chloride tetrasulphonate based photosensitizer on lung cancer cells. J Photochem Photobiol B Biol. May 2019;194:96–106. 10.1016/J.JPHOTOBIOL.2019.03.018. 10.1016/j.jphotobiol.2019.03.018 30953915

[53] Crous A, Sundar S, Kumar D, Abrahamse H. Effect of dose responses of hydrophilic aluminium (III) phthalocyanine chloride tetrasulphonate based photosensitizer on lung cancer cells, *J. Photochem. Photobiol. B Biol*, vol. 194, no. November 2018, pp. 96–106, 2019, 10.1016/j.jphotobiol.2019.03.018. 10.1016/j.jphotobiol.2019.03.018 30953915

[54] Tynga IM, Houreld NN, Abrahamse H (2013). The primary subcellular localization of Zinc phthalocyanine and its cellular impact on viability, proliferation and structure of breast cancer cells (MCF-7). J Photochem Photobiol B Biol.

[55] Ndhundhuma I, Hauser C, Scalfi-Happ C, Rück A, Steiner R (2011). Subcellular co-localization of aluminum (III) phthalocyanine chloride tetrasulphonate with fluorescent markers in the human melanoma cell-line HT-144. Med Laser Appl.

[56] Mokoena DR, George BP, Abrahamse H. Photodynamic therapy induced cell death mechanisms in breast cancer. Int J Mol Sci. 2021;22(19). 10.3390/ijms221910506. 10.3390/ijms221910506 PMC8508861 34638847

[57] Haywood-Small SL, Vernon DI, Griffiths J, Schofield J, Brown SB (2006). Phthalocyanine-mediated photodynamic therapy induces cell death and a G0/G1 cell cycle arrest in cervical cancer cells. Biochem Biophys Res Commun.

[58] Eom YW (2005). Two distinct modes of cell death induced by doxorubicin: apoptosis and cell death through mitotic catastrophe accompanied by senescence-like phenotype. Oncogene.

[59] Rahman MM, Khan MA. Anti-cancer potential of south Asian plants. Nat Prod Biopros. Jun. 2013;3(3):74–88. 10.1007/s13659-013-0027-6.

[60] Sandler E et al. Nov., Efficacy of ifosfamide and doxorubicin given as a phase II ‘window’ in children with newly diagnosed metastatic rhabdomyosarcoma: A report from the Intergroup Rhabdomyosarcoma Study Group*, *Med. Pediatr. Oncol*, vol. 37, no. 5, pp. 442–448, 2001, 10.1002/MPO.1227. 10.1002/mpo.1227 11745872

[61] Aziz B, Khurshid A, Ahmat L, Khan JA, Alam M, Ikram M (2023). In vitro evaluation of the cytotoxic potential of Ficus palmata and its combination with chemotherapy and photodynamic therapy. Laser Phys Lett.

[62] Broekgaarden M, et al. Neoadjuvant photodynamic therapy augments immediate and prolonged oxaliplatin efficacy in metastatic pancreatic cancer organoids. Oncotarget. Feb. 2018;9:13009. 10.18632/ONCOTARGET.24425. 10.18632/oncotarget.24425 PMC5849191 29560127

[63] Ahn JC, Kang JW, Shin JI, Chung PS (2012). Combination treatment with photodynamic therapy and curcumin induces mitochondria-dependent apoptosis in AMC-HN3 cells. Int J Oncol.

[64] Xin J (2018). Comparison of the synergistic anticancer activity of AlPcS4 photodynamic therapy in combination with different low-dose chemotherapeutic agents on gastric cancer cells. Oncol Rep.

[65] Sci-Hub | Recent Advances in Nanomaterial-Assisted Combinational Sonodynamic Cancer Therapy. Advanced Materials, 32(47), 2003214 | 10.1002/adma.202003214, Accessed: Jan. 08, 2023. [Online]. Available: https://sci-hub.st/https://onlinelibrary.wiley.com/doi/abs/10.1002/adma.202003214?casa_token=YGnvDYbJu6AAAAAA:7XKfAPhwb00SEvn_sS_R01B13F8eoqqcW4I7burAHU-P8waj6KG5b8iNgrJF3GXFdHiG_r_qIDp7nrju. 10.1002/adma.202003214 33064322

[66] Muniyandi K, George B, Parimelazhagan T, Abrahamse H. Molecules role of Photoactive Phytocompounds in Photodynamic Therapy of Cancer, 10.3390/molecules25184102. 10.3390/molecules25184102 PMC7570746 32911753

[67] Mokhtari RB, Homayouni TS, Baluch N. COMBINATION THERAPY IN COMBATING CANCER, 8, 23, pp. 38022–43, 2017. 10.18632/oncotarget.16723 PMC5514969 28410237

[68] Ahmad KS, Kiani BH, Mehmood A, Iqbal MS, Azad Kashmir EVALUATION OF ANTICANCER ACTIVITIES OF SELECTED MEDICINAL PLANTS FROM GANGA CHOTI. March, Evaluation of anticancer activities of selected medicinal plants from Ganga Choti, lesser Himalaya, Bagh, LESSER HIMALAYA, BAGH, AZAD KASHMIR, vol. 4, no. 2021, 10.30848/PJB2021-4(38)CITATIONS.

